# First person – Sheeza Mughal

**DOI:** 10.1242/dmm.050778

**Published:** 2024-04-24

## Abstract

First Person is a series of interviews with the first authors of a selection of papers published in Disease Models & Mechanisms, helping researchers promote themselves alongside their papers. Sheeza Mughal is first author on ‘ Taurine activates the AKT–mTOR axis to restore muscle mass and contractile strength in human 3D *in vitro* models of steroid myopathy’, published in DMM. Sheeza is a PhD Student in the lab of Javier Ramón-Azcón at the Institute for Bioengineering of Catalonia (IBEC) in Barcelona, Spain, and is interested in developing disease models to simplify understanding of otherwise complex diseases.


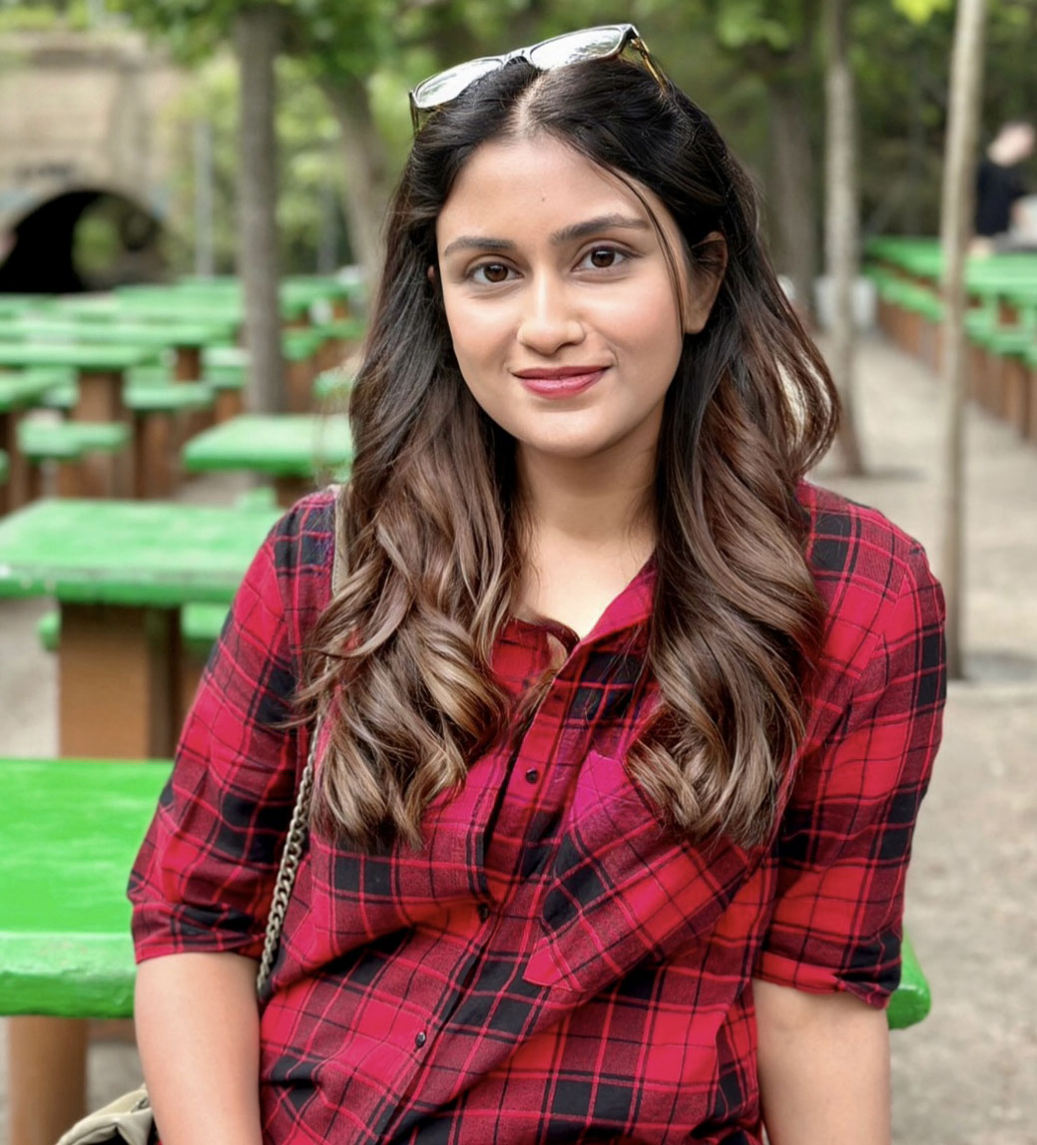


**Sheeza Mughal**

**How would you explain the main findings of your paper to non-scientific family and friends?**

Imagine our muscles as a complex system of building blocks that can be affected by certain medications or health conditions. In our study, we created a miniature version of these human muscle systems in the lab to understand how steroids, specifically dexamethasone, can cause muscle problems. We found that when we treated our lab-grown muscles with dexamethasone, it led to significant weakening and shrinkage of the muscle fibers, similar to what happens in a condition called steroid myopathy. This is a challenge often faced by people who use corticosteroid medications for a long time or have certain tumors.

To understand what happens at a deeper level, we discovered that a protein called glucocorticoid receptor moves into the cell nucleus and another system called the ubiquitin–proteasome system gets activated. These changes work together to break down the muscle tissue, causing it to lose strength and size.

Now, the exciting part is that we explored a potential solution to this problem. We tested a substance called taurine on our lab-grown muscles and found that it could counteract the negative effects of dexamethasone. Taurine seemed to boost a pathway called AKT–mTOR, which helps in maintaining muscle strength and size.

One important takeaway from our study is that simply stopping the use of the corticosteroid medication is not enough to restore muscle health. However, when taurine was used alongside the medication, it significantly improved muscle strength and its ability to build proteins, suggesting a potential treatment approach for individuals dealing with corticosteroid use or who have adrenal tumors.

In summary, our research not only helps us understand why certain medications or health conditions affect muscles, but also points towards a promising solution involving taurine to improve muscle health in these situations.

This study underscores the long-term effects of corticosteroid use on muscles, emphasizing the need for proactive interventions beyond discontinuation of medication.

**What are the potential implications of these results for your field of research?**

The results of this research carry significant implications for advancing our understanding and treatment of muscle-related disorders, particularly in the context of steroid myopathy and corticosteroid use. The identification of taurine as a promising therapeutic agent highlights the potential for targeted interventions to alleviate the detrimental effects of corticosteroid-induced muscle damage. The elucidation of molecular and functional aspects of steroid myopathy using a bioengineered 3D muscle model in this study contributes valuable insights into the underlying mechanisms of muscle atrophy. The concept of combinatorial treatment approaches, involving taurine alongside corticosteroid medications, opens new avenues for enhancing muscle health and function. Furthermore, this study underscores the long-term effects of corticosteroid use on muscles, emphasizing the need for proactive interventions beyond discontinuation of medication. The developed muscle model serves as a versatile tool for translational research, offering a controlled environment to explore potential therapeutics for various muscle disorders. Overall, these findings have broad implications for advancing targeted therapies, improving clinical practices and fostering innovation in the field of muscle research using 3D *in vitro* bioengineered tissue/organoid models.

**What are the main advantages and drawbacks of the experimental system you have used as it relates to the disease you are investigating?**

The advantages of tissue-on-chip platforms are that they enable replacement of animals as experimental models and offer a microphysiological environment similar to the *in vivo* environment. Organoids or tissue models bioengineered from human cells, such as those shown in this research, allow procuring data specific to the human species. They also allow development of patient-centered models using either patient cells or fluids.

One notable disadvantage is the complexity and heterogeneity of different organs and tissues, making it difficult to create a universal platform that accurately mimics the diverse microenvironments of the human body. Additionally, the current state of organ-on-chip technology often focuses on individual organs or tissues, lacking the capability to fully emulate the intricate interplay between multiple organs in a systemic context. Moreover, there are limitations in terms of scalability, reproducibility and standardization across different organ-on-chip devices and laboratories.

**Figure:**
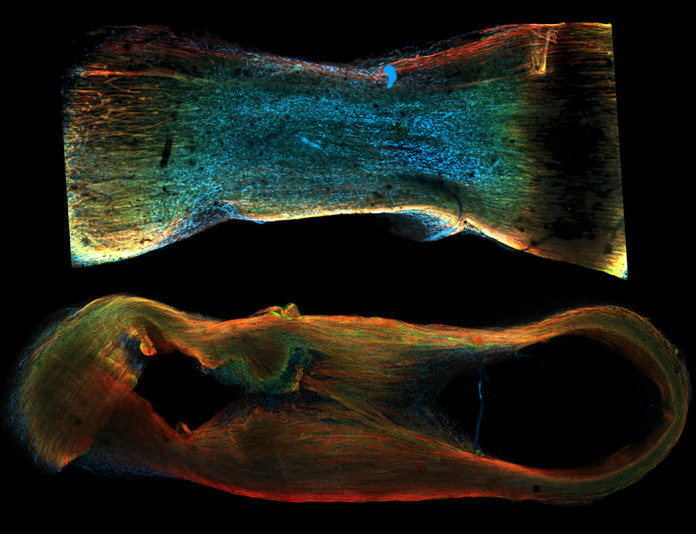
Representative immunofluorescence image of a mature bioengineered muscle tissue stained for sarcomeric actinin, F-actin and nuclei.

**What has surprised you the most while conducting your research?**

Without careful monitoring, treatment for one health concern may be damaging to another organ system. The answer to this may not always be as simple as an optimization of dosages.

**What do you think is the most significant challenge impacting your research at this time and how will this be addressed over the next 10 years?**

Understanding all facets of health, disease, their diagnoses and therapies is a lifelong process that requires a stable footing. At the moment, lack of career certainty (with regards to contractual positions) is perhaps the biggest challenge impacting my research. In the next 10 years, with improved career and financial stability, this problem might be addressed, giving me a better chance to continue research.

**What changes do you think could improve the professional lives of scientists?**

Lack of financial stability and career certainty are the biggest bottlenecks for both early- and mid-career scientists. Measures focusing on respectable and livable remuneration packages are especially needed to help achieve work-life balance. Moreover, lack of clarity in academic and research trajectories may also (and often do) force many talented minds to change career paths in favor of more lucrative alternatives. Without measures that ensure quality living, achieving quality research becomes a gamble.

**What's next for you?**

As a third-year PhD student, I am focusing on understanding health conditions that are often neglected or not given due attention. Next, I aim to explore the implications of mitochondrial dysfunction on peripheral fatigue with real-time evaluation and proposition of possible therapeutic measures.
